# Teaching Application of 3D-printed Models for Nasal Analysis

**DOI:** 10.1097/GOX.0000000000006149

**Published:** 2024-09-09

**Authors:** Nneoma S. Wamkpah, Robert T. Cristel, Zachary O’Connor, Andrea Hanick, Dennis Nguyen, John J. Chi

**Affiliations:** From the * Department of Otolaryngology—Head and Neck Surgery, Washington University in St. Louis, St. Louis, Mo.; † Facial Plastic and Reconstructive Surgery, Department of Otolaryngology—Head and Neck Surgery, Washington University School of Medicine, St. Louis, Mo.; ‡ Tech Den & Student Technology Services, Washington University Information Technology, Washington University School of Medicine, St. Louis, Mo.; § Missouri Ear, Nose and Throat Center, Columbia, Mo.; ¶ Plastic and Reconstructive Surgery, Department of Surgery, Washington University School of Medicine, St. Louis, Mo.

## Abstract

**Background:**

A major challenge in learning rhinoplasty is correlating patients’ external and internal nasal structures. We aim to explore the application of three dimensional (3D)-printed models of nasal bony-cartilaginous structures in identifying accurate nasal anatomy.

**Methods:**

Otolaryngology—head and neck surgery and plastic and reconstructive surgery residents matched patient photograph models, described relative nasal bony-cartilaginous anatomy, completed pre- and postactivity self-evaluations (based on otolaryngology “nasal deformity” milestones including “anatomy,” “function,” “aesthetic,” and “etiology”), and rated the 3D-printed models’ usefulness. Descriptive statistics were measured.

**Results:**

Thirty-seven residents correctly matched four of six model-photograph pairs and correctly described 15 of 30 anatomic relationships, on average. There was a moderate, statistically significant correlation between postgraduate year and number of correctly matched model-photograph pairs (Spearman rho = 0.58, 95% CI 0.24–0.79) and total items correct (Spearman rho = 0.61, 95% CI 0.28–0.81). Self-ratings on milestones decreased postexercise in all subcategories except “function.” From 0 (low) to 100 (high), learners found the exercise useful (median 85 of 100) with a high recommendation for future use (median 87 of 100).

**Conclusions:**

Three-dimensional printed models are a valuable tool for understanding nasal anatomy. Continued standardization of designs and assessments of their educational utility will enhance their broader dissemination and implementation.

**Takeaways**

**Question:** Can three-dimensional (3D) printed models of nasal anatomy be used for understanding nasal analysis?

**Findings:** Resident participants reported 3D-printed models to be both highly useful and recommended for learning to describe patient-specific internal nasal anatomy. Residents with greater clinical experience performed significantly better on matching patients to their specific 3D-printed model, but both senior and junior resident groups performed similarly when analyzing the position of specific nasal structures.

**Meaning:** Three-dimensional printed models of nasal anatomy can be a valuable educational tool by providing a patient-specific visual correlate with standardized rhinoplasty photographs in a safe simulation environment.

## INTRODUCTION

Rhinoplasty is one of the most complicated and intricate surgical procedures to learn. Each procedure must be individualized to a patient’s anatomy and surgical plans must be accurately made based on the preoperative evaluation; however, the underlying nasal framework is only exposed at the time of surgery, and there are a multitude of structural variances with which any patient could present. Adding to these challenges in correlating patients’ external nasal examination with their internal anatomy are discrepancies in measuring competency at the resident level, as learners may perceive their own performance during rhinoplasty differently from how attending surgeons rate them.^1^

Simulation-based learning has blossomed and refined over the past few decades within surgical subspecialty training, including plastic surgery of the head and neck, by providing opportunities for practicing physical examination and technical skills in safe, less stressful environments outside of clinical spaces, such as the operating room.^2–7^ Potential benefits are improved operating room performance and efficiency for residents through repeated practice, which could impact “hidden” costs of prolonged operative times or technical medical errors.^6–8^ Within both otolaryngology and plastic surgery, creative simulators have been demonstrated using low-cost materials to explore competency-based surgical education compared with traditional surgical training.^7,8^ An example of simulation-based learning is using models created from three-dimensional (3D) printing technology. Three-dimensional printing has the benefits of creating personalized, complex simulators that are malleable to meet educational gaps, patient-specific and anatomically accurate,^9,10^ and rapidly manufacturable.^11^ These features make 3D-printed models ideal tools for rhinoplasty education and learning the nuances of individual patient anatomy.^12–16^

After completion of simulation-based training or real-life surgical performances, residents benefit from feedback using standard measures of competency. For a multitude of reasons (limited work duty hours and intraoperative autonomy, medicolegal and patient safety issues), a growing concern exists over the perceived lack of confidence and competence among graduating residents, resulting in increased interest in developing valid and reliable evaluation metrics.^17^ The otolaryngology milestones were developed by an expert group of otolaryngologists and reviewed by members of the Accreditation Council of Graduate Medical Education as a way for otolaryngology residents to track their readiness for unsupervised clinical practice in various subspecialty disease processes (milestones) and Accreditation Council of Graduate Medical Education core competencies (systems-based practice, practice-based learning and improvement, professionalism, and interpersonal and communication skills).^18,19^ Residents and the attendings who evaluate them provide a rating of 1 to 5 for each milestone, with 4 as the target competency level for graduation and 5 as an aspirational level not everyone achieves by the end of residency.^18^ Through ratings on milestones, residents gain introspection about their confidence with understanding and managing pathologic processes via quantitative metrics that potentially change with time or experience.

Although the use of 3D-printed models in rhinoplasty training is not entirely unique,^6,7,10^ this study aims to assess the feasibility of 3D-printed models in facilitating resident understanding of underlying preoperative nasal framework for patients undergoing rhinoplasty and to measure residents’ confidence with the rhinoplasty physical examination, anchored on the otolaryngology milestones for “nasal deformity.”^18^ We hypothesized that residents would find the 3D-printed models to be an effective educational tool for anticipating underlying nasal bony and cartilaginous nasal anatomy in patients who underwent rhinoplasty.

## METHODS

This was a single-institution cohort study of the application of 3D-printed models as an educational tool for residents performing nasal analysis for rhinoplasty and was approved by the Washington University institutional review board (IRB #201812125). Two populations were recruited. The first were patients who presented for rhinoplasty or septorhinoplasty and had preoperative computed tomography (CT) scans, who gave written consent for the use of their photographs for the study. The second included residents in the Washington University Department of Otolaryngology—Head and Neck Surgery (OHNS) and Division of Plastic and Reconstructive Surgery (PRS) who gave verbal consent to participate in the study.

### Creation of 3D-printed Models

**Fig. 1.**
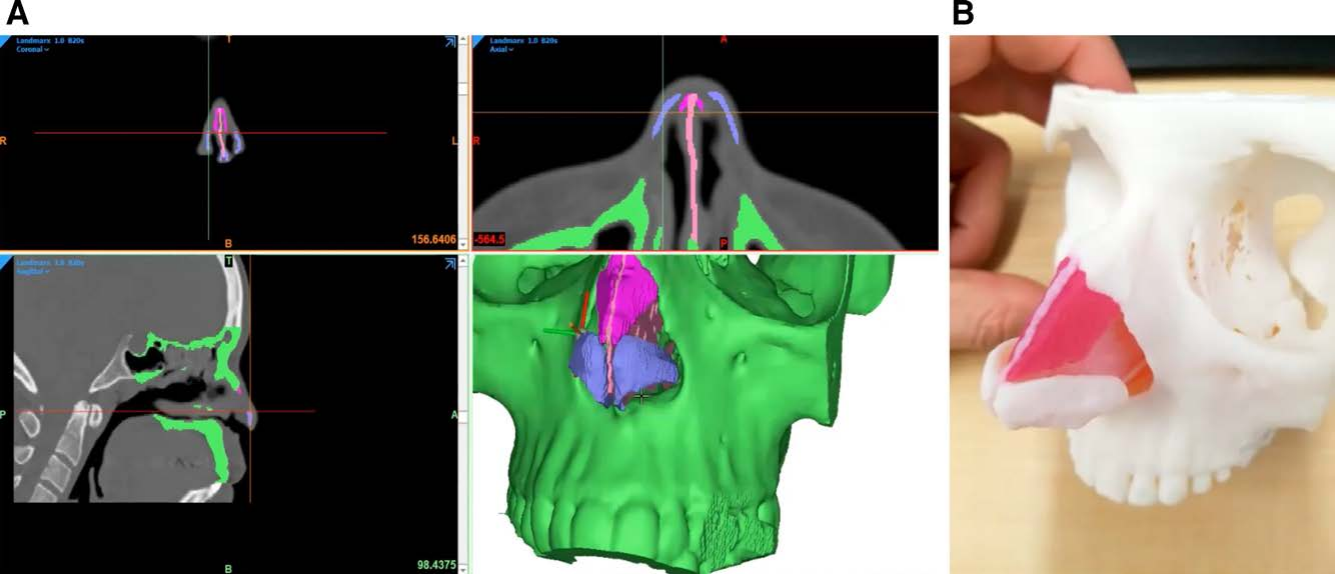
Data segmentation via software prior to 3D printing. A, After obtaining rhinoplasty photographs, datasets were imported into Materialise Mimics, a 3D medical image segmentation software. The bone threshold was used for the initial segmentation and then manually fine-tuned. The cartilaginous structures’ masks were all added manually, based off imaging, photography, and physician guidance. B, The models were printed on Stratasys J750 PolyJet technology using the Vero materials, the support material was removed through pressure washing, and quality assurance was checked to make sure the printed models were accurate to the digital models.

Standard rhinoplasty photographs (frontal, left and right lateral, worm’s and bird’s eye views) were obtained from patient participants. Deidentified multislice CT scans of these patients were used with the photographs to generate patient-specific mutually exclusive 3D-printed models (Fig. 1). The CT datasets were imported into Materialise Mimics, a 3D medical image segmentation software, to start the creation process of going from medical imaging to 3D-printed model. The structures of interest for segmentation were the nasal bones; surrounding midfacial bones; and nasoseptal, upper lateral, and lower lateral cartilages. The bone threshold was used initially to segment the facial and nasal bone mask and then manually fine-tuned. The septum, upper, and lower lateral cartilage masks were all added manually, based on patients’ photographs and physician guidance (A.H. and J.J.C.). Once the segmentation masks were deemed viable, they were converted to surface tessellation language (.STL) file meshes and exported into Materialise 3-matic for final editing and preparation for printing. Materialise 3-matic is a computer-aided design software where final adjustments are made, such as debris removal, smoothing, wall thickening, model identifiers, and the preparation of the models for 3D printing. With the final .STL files ready, all models were imported into GrabCAD Print, the slicing software for setting up the files to send to the 3D printer. The models were printed on Stratasys J750 polyjet technology using the Vero materials for each structure to have its own assigned color. Once printed, the support material was removed through pressure washing, and quality assurance of the models was checked to ensure fidelity with the CT and photograph digital images.

### Resident Learning Activity: Matching and Nasal Analysis

Residents were presented with six 3D-printed models and six sets of standardized rhinoplasty photographs. Responses were recorded on a REDCap^20,21^ survey containing a multiple-choice format for the matching and labeling exercises, a visual analog scale (VAS) format for self-evaluations on nasal deformity milestones, and an open-ended format for additional comments.

**Fig. 2.**
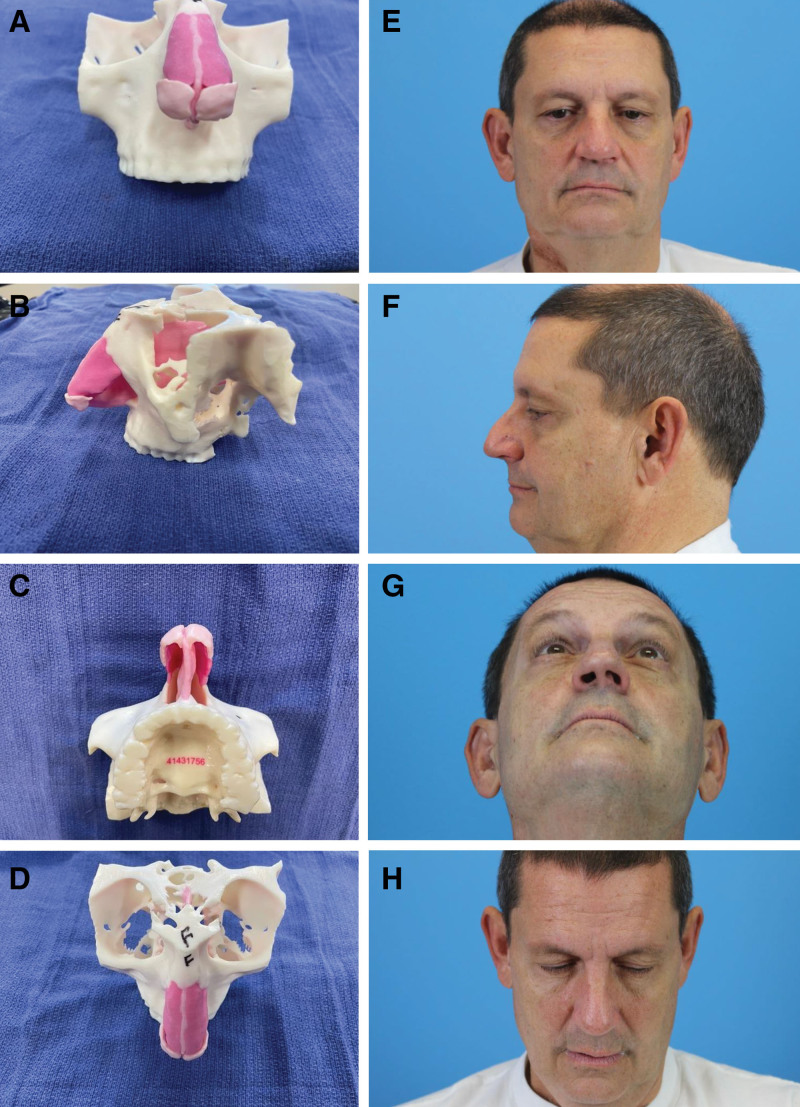
Case example of a patient’s standard rhinoplasty photographs and his patient-specific mutually exclusive 3D-printed model. The model is seen from frontal (A), left lateral, (B) worm’s (C) and bird’s eye (D) views. E-H, The patient seen from the corresponding views.

First, they matched each of the 3D-printed models to a patient by comparing the model with the rhinoplasty photographs; an example of a study patient is shown in Figure 2. Immediately after matching the model-photograph pairs, residents were tasked to label the anatomic direction (midline, deviated to patient’s left or right) of five internal nasal structures (nasal bones, dorsal septum, caudal septum, upper lateral cartilage, and lower lateral cartilage) on each patient by looking at the rhinoplasty photographs; they could also utilize the 3D-printed models (after matching) to guide their choices. The residents were blinded to the correct answers until both matching and nasal analyses were completed. Each resident performed the exercise independently of one another, with responses saved on their individual REDCap survey forms.

### Resident Self-evaluation

Before and after the exercise, residents rated their ability to confidently assess nasal “anatomy,” “function,” “aesthetic,” and “etiology”—broad subcategories featured on the otolaryngology nasal deformity milestones^18^ using a VAS ranging from 0 (minimum) to 100 (maximum). [**See figure, Supplemental Digital Content 1,** which displays how otolaryngology nasal deformity milestones were broadly defined as four conceptual categories (anatomy, function, aesthetic, and etiology). Residents self-rated their confidence in performing these items on a VAS from 0 (minimum) to 100 (maximum). http://links.lww.com/PRSGO/D489.] Finally, residents were asked to rate the utility of the 3D-printed model exercise and whether they would recommend the exercise for future use on a VAS ranging from 0 (low) to 100 (high). [**See figure, Supplemental Digital Content 2,** which displays how residents were asked to rate the utility and how likely they would recommend this exercise using a VAS from 0 (minimum) to 100 (maximum). http://links.lww.com/PRSGO/D490.]

### Statistical Analysis

For patient participants, demographic variables (age, sex, and self-identified race) were analyzed with descriptive statistics. Descriptive variables for resident participants included self-identified gender, residency program, postgraduate year, and experience level with nasal surgery (number of either rhinoplasty or septoplasty courses attended, or number of rhinoplasty and septoplasty procedures performed as either an assistant surgeon or resident surgeon). To explore differences in performance across training experience levels, residents were stratified into “junior resident” and “senior resident” categories corresponding to postgraduate years (PGY) 1–3 and 4–6, respectively. Association between resident experience and demographic variables were measured with the Mann-Whitney U test for continuous outcomes and χ^2^ or Fisher exact test for categorical outcomes.

The primary outcome was resident performance on matching model-photograph pairs and correctly describing the position of the five internal nasal structures. Each correct model-photograph pair was scored one point (total six points), and each correctly analyzed nasal structure was scored one point (total 30 points). Descriptive statistics reported average scores on each part of the exercise. The strength of association between matching performance and PGY level was measured as the median or proportion difference and the corresponding 95% CI. Correlations between performance on the matching and nasal analysis exercises and resident experience level (number of rhinoplasty/septoplasty cases performed) were calculated (Spearman rho). The secondary outcomes were resident pre-post self-evaluations on the otolaryngology nasal deformity milestones and ratings of this exercise’s utility; the median values of these ratings were described.

All statistical analyses were performed at a two-sided alpha level of 0.05. Analyses were conducted on SPSS Statistics version 28 (IBM Corporation, Armonk, N.Y.). This was a feasibility study applying 3D-printed models for residents learning nasal analysis; thus, we planned to analyze a convenience sample of OHNS and PRS residents.

## RESULTS

Six patients, 50% of whom were male (n = 3) with a median (minimum-maximum) age of 40 (17–59) years old, constituted the patient study population. One patient self-identified as African American, and the rest as White. All had sustained nasal trauma, thus had CT head or face scans obtained as part of their trauma workup elsewhere, before evaluation for nasal surgery. On average, each model cost $550 and took 12–16 hours to create.

Thirty-seven residents completed the study (Table 1); 23 were OHNS residents and 14 were PRS residents. Most were male (n = 21, 56.8%). There was a balanced distribution of residents in each postgraduate year. Most had participated in a facial plastic surgery or craniofacial surgery rotation in residency (n = 25, 67.6%) and had performed at least one rhinoplasty (n = 24, 64.9%) or septoplasty (n = 25, 67.6%). Almost half of the residents had not attended a rhinoplasty or sinus surgery course (n = 18, 48.6%). Statistically significant differences between junior- and senior-level residents existed for participation in a facial plastic surgery/craniofacial surgery rotation and for the number of rhinoplasty and septoplasty cases performed, demonstrating that exposure to surgical experiences was related to the time spent in residency.

**Table 1.** Description of Resident Participants

	Residency Level, n (%)		*P*
	Junior Resident, n* *= 22	Senior Resident, n = 15	
Male sex	12 (54.5)	9 (60)	0.742
Residency program			0.823
Otolaryngology	14 (63.6)	9 (60.0)	
Plastic surgery	8 (36.4)	6 (40.0)	
Participated in a facial plastic surgery or craniofacial surgery rotation	10 (45.5)	15 (100)	**0.001** *
No. rhinoplasty cases performed			
None	13 (59.1)	0 (0)	**0.001** †
1–2	5 (22.7)	0 (0)	
3–5	3 (13.6)	4 (26.7)	
6–8	1 (4.5)	5 (33.3)	
9+	0 (0)	6 (40.0)	
No. septoplasty cases performed			
None	12 (54.5)	0 (0)	**0.001** †
1–2	6 (27.3)	0 (0)	
3–5	2 (9.1)	3 (20.0)	
6–8	1 (4.5)	1 (6.7)	
9+	1 (4.5)	11 (73.3)	
No. rhinoplasty/sinus surgery courses attended, median (min−max)	0 (0**–**5)	1 (0**–**6)	0.174†
No. correctly matched model-photograph pairs	3 (0**–**6)	6 (3**–**6)	**0.001** †
No. correct items on nasal analysis, median (min−max)	14 (8**–**22)	17 (6**–**23)	0.176†

Bold values are statistically significant.

* Fisher exact test.

† Mann-Whitney U test.

Max, maximum; Min, minimum.

In total, the median number of correctly matched model-photograph pairs was four (of six) and the median number of correctly labeled nasal analysis items was 15 (of 30). When subdivided by PGY level, senior residents had a statistically significantly better performance than junior residents in identifying correct model-photograph pairs (*P* < 0.001) but not in labeling correct relationships of nasal bony and cartilaginous structures (*P* = 0.176) (Table 1). There was a moderate and statistically significant correlation between the number of correctly matched model-photograph pairs and the number of rhinoplasty cases performed [Spearman rho (95% CI) = 0.62 (0.36–0.79), *P* < 0.001].

**Fig. 3.**
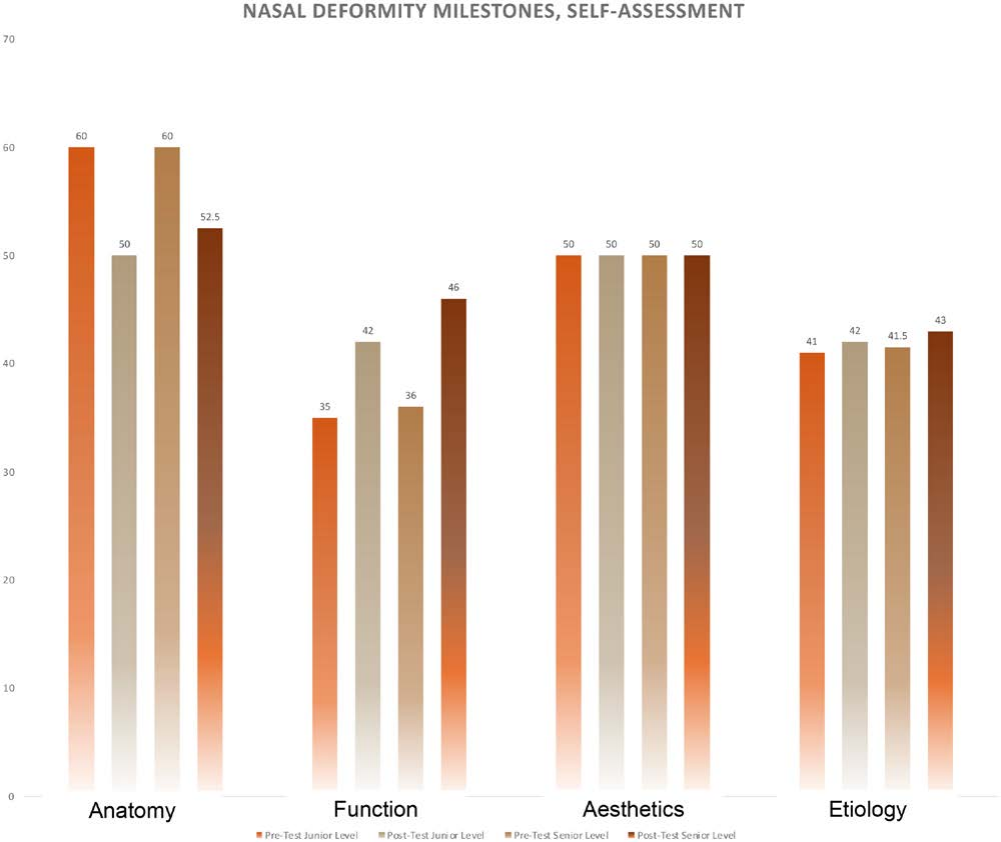
Bar chart illustrating resident self-evaluation of their confidence in performing the otolaryngology nasal deformity milestones, before and after the 3D-printed model exercise.

Residents rated their confidence using the otolaryngology nasal deformity milestones, which were broadly summarized into four categories: anatomy, function, aesthetics, and etiology (Table 2, Fig. 3). The median differences between pre- and postexercise self-ratings were not statistically significant in any milestone category. In almost every milestone category, most residents rated themselves the same or lower postexercise, except for the function category—residents rated themselves higher postexercise. Junior-level residents rated themselves significantly lower on average compared with senior-level residents in all categories except for aesthetics.

**Table 2.** The Median Difference (Minimum-Maximum) in Self-evaluation of Confidence with the Otolaryngology Nasal Deformity Milestones, Stratified by Residency Experience Level

	PGY 1–3 (n = 22)	PGY4–6 (n = 15)
Anatomy	−1.0 (−21 to 100)	0 (−20 to 20)
Function	3 (−12 to 48)	0 (−20 to 38)
Aesthetic	0 (−22 to 80)	0 (−15 to 35)
Etiology	0 (−40 to 50)	−15 (−24 to 19)

The residents found the exercise to be useful (median VAS rating 85 of 100) and were highly likely to recommend its use in the future (median VAS rating 87 of 100). Optional, free-text comments provided by the residents after completing the exercise were positive (Table 3).

**Table 3.** Residents Provided Additional Optional Comments after Completing the Exercise

Otolaryngology, PGY-1	“This was an excellent demo! I think it would be very helpful to have direct teaching opportunities with 3D nasal models as part of our curriculum.”
Otolaryngology, PGY-3	“[I] realized how much I did not notice when specifically asked to evaluate each structure.”
Otolaryngology, PGY-4	“This was a good exercise, and I would like to use 3D models to help me correlate my physical exam/facial analysis skills in the future.”
Otolaryngology, PGY-5	“[The exercise was] educational in and of itself.”
PRS, PGY-6	“[This exercise was] a great way to do this…with the pictures and models; [makes you] look at small details. [This was] fun.”

## DISCUSSION

The role of 3D-printed models in surgical education is still being refined. In our study, OHNS and PRS residents with higher experience levels performed better in identifying the overall nasal structural abnormality of model-photograph pairs, but residents at both senior and junior levels performed similarly on analysis of independent nasal structures. Senior-level residents, through experience, may gain a gestalt for the composite anatomy and thus have success matching model-photograph pairs, but residents may not fully understand the specific underlying nasal anatomy responsible for patients’ appearance.

Postexercise, residents’ self-ratings on the otolaryngology nasal deformity milestones (Fig. 3) stayed the same or were lower for anatomy, aesthetic, and etiology categories. Self-ratings improved for the function category. Our study was not powered to show a statistically significant median difference in pre- versus postexercise self-ratings in any of the four categories. However, based on the trends shown, perhaps upon completion of the exercise, residents were made aware of individual gaps in their knowledge, hence reported a lower confidence in certain aspects of the nasal physical examination. In fact, multiple residents commented that this exercise was a revelation to them about their level of certainty in understanding nasal analysis (Table 3). This suggests that 3D-printed models could serve as a pre- and posttest barometer of resident confidence in nasal surgery knowledge, potentially as an adjunct to targeted didactic lessons and surgical exposure to rhinoplasty. A similar finding was noted in a study by Gupta et al,^13^ which used 3D-printed models with a simultaneous didactic session to reinforce anatomical concepts. After the combined didactic and practical lessons in the study by Gupta et al,^13^ residents self-rated an improved understanding of the goals of rhinoplasty, approach to the nasal septum, osteotomies, grafts, and changes to the tip/appearance/nasal airway.^13^ A recently published report used a 3D-printed model of a normal patient combined with a brief lecture in the setting of an academic national meeting to teach osteotomy techniques to attending surgeons, fellows, residents, and medical students.^22^ In these studies, improvements in understanding are challenging to tease out as solely being associated with the 3D-printed models, the didactics, or both. In comparison, our study focused on using 3D-printed models in isolation. Another key difference in our study was the focus on evaluating comprehension of foundational principles of the rhinoplasty physical exam anchored on validated otolaryngology milestones, whereas other published studies described teaching anatomy^13^ but measured confidence in understanding surgical principles and used unvalidated surveys.^13,22^ In this study, results were isolated to use of a 3D-printed model without accompanying other educational material. Additionally, there was no standard time limit—a resident could take as little or as much time in doing the exercise as they wished, and some residents may have gone through the exercise more quickly if they had other competing interests (going to their assigned operating room cases or finishing up patient clinical documentation, etc). Further studies comparing identification of underlying bony and cartilaginous structures using a 3D-printed model head-to-head against using other types of learning practices (ie, audiovisual material), or comparing performance under varying time exposures will need to be adequately powered to detect a difference between pre- and postexercise self-ratings.

Overall, feedback about our exercise was positive among the residents. No resident mentioned that the exercise took up a significantly burdensome amount of time or difficulty to complete. Most also recommended the exercise to be officially incorporated into resident training. Thus, this study supports the hypothesis that 3D-printed models may be a beneficial supplement to didactic and hands-on rhinoplasty education, consistent with published literature.^7,13,22^

This was a feasibility study of the teaching application of 3D-printed models in nasal surgery. Although innovative, inclusive of interdisciplinary training programs, and structured on rigorously vetted otolaryngology milestones, this study has a few limitations. First, as a single-institution study, there is volunteer bias and a lack of generalizability to other academic centers, which may have different opportunities for rhinoplasty or septoplasty surgical experiences. With a focus on characterizing underlying bony and cartilaginous framework, the 3D-printed models in our study lacked an intranasal mucosal layer and external skin and soft tissue layers, thus differing from composite models previously described.^13–16,22^ Other published 3D-printed rhinoplasty models created stereolithographic models or surgical guides using 3D-printed photography,^6,9^ which differed from our model that was created from CT imaging and two-dimensional photography. However, given the emphasis on understanding underlying bony/cartilaginous deformity, we felt our strategy for developing the models in this study was appropriate. Blinding the residents to the correct answers to the model-photograph matching portion of the exercise may have affected their performance on the nasal analysis portion of the exercise if they proceeded with inaccurate information while trying to describe the nasal structures.

In the future, the next step would be to develop a supplementary targeted teaching guide (including didactic and audiovisual material) with the 3D-printed models and measure their combined ability to enhance resident educational experience, or perhaps serve as a substitute or complement to real-life surgical experiences, including comparative cost and time data to traditional surgical training.

## CONCLUSIONS

Three-dimensional printed models are valuable educational tools for teaching underlying nasal anatomy. These models help correlate patient-specific external nasal examination to internal nasal anatomy and are recommended by surgical residents. The nascent field of 3D technology in patient care and resident education has become increasingly refined but still needs validated measures to track its efficacy and standardization to promote its dissemination and implementation.

## DISCLOSURES

The authors have no financial interest to declare in relation to the content of this article. This publication [or project] was supported in whole or in part by the Mallinckrodt Institute of Radiology at the Washington University School of Medicine, The Foundation for Barnes-Jewish Hospital and their generous donors, and the Washington University Institute of Clinical and Translational Sciences which is, in part, supported by the NIH/National Center for Advancing Translational Sciences, Clinical and Translational Science Award grant #UL1 TR002345.

## PATIENT CONSENT

*The patient provided written consent for the use of his image.*

## Supplementary Material


