# Supplementary material for: Teaching Application of 3D-printed Models for Nasal Analysis
Source: Plast Reconstr Surg Glob Open. 2024 Sep 9;12(9):e6149. doi: 10.1097/GOX.0000000000006149 (PMC11383718; doi:10.1097/GOX.0000000000006149)
Supplement: Supplementary file 1 [file gox-12-e6149-s001.pdf]

SDC 1.

On a scale from 0-100, please indicate your level of confidence with performing each of the procedures below:

- *Demonstrate understanding of normal nasal anatomy*
- *Perform comprehensive dynamic nasal function analysis*
- *Identify aesthetic/cosmetic abnormalities*
- *Correlate examination findings with underlying structural etiologies*
