# Supplementary material for: Teaching Application of 3D-printed Models for Nasal Analysis
Source: Plast Reconstr Surg Glob Open. 2024 Sep 9;12(9):e6149. doi: 10.1097/GOX.0000000000006149 (PMC11383718; doi:10.1097/GOX.0000000000006149)
Supplement: Supplementary file 2 [file gox-12-e6149-s002.pdf]

SDC 2.

On a scale from 0-100, please rate the following:

- *Working with the 3D models was a useful experience.*
- *The 3D rhinoplasty models should be recommended for any resident to improve his/her skill at nasal analysis.*
